# Impact of type 2 diabetes mellitus on left ventricular deformation in non-ischemic dilated cardiomyopathy patients assessed by cardiac magnetic resonance imaging

**DOI:** 10.1186/s12933-022-01533-5

**Published:** 2022-06-04

**Authors:** Meng-Ting Shen, Yuan Li, Ying-Kun Guo, Li Jiang, Yue Gao, Rui Shi, Zhi-Gang Yang

**Affiliations:** 1 Department of Radiology, West China Hospital, Sichuan University, No.37 Guoxue Xiang, Chengdu, 610041 Sichuan China; 2 Department of Radiology, Key Laboratory of Birth Defects and Related Diseases of Women and Children of Ministry of Education, West China Second University Hospital, Sichuan University, 20# South Renmin Road, Chengdu, 610041 Sichuan China

**Keywords:** Type 2 diabetes mellitus, Dilated cardiomyopathy, Strain, Magnetic resonance imaging, Heart failure

## Abstract

**Background:**

Type 2 diabetes mellitus (T2DM) increases the risk of worse long-term outcomes in patients with non-ischemic dilated cardiomyopathy (NIDCM). However, the additive effects of T2DM on left ventricular (LV) function in NIDCM remain unclear. Accordingly, we aimed to investigate the impact of comorbid T2DM on LV deformation in NIDCM individuals.

**Materials and methods:**

Three hundred forty-two NIDCM patients without T2DM [NIDCM (T2DM−)], 93 with T2DM [NIDCM (T2DM+)] and 80 age- and sex-matched normal controls who underwent cardiac magnetic resonance scanning were included. LV geometry, function, and LV global strains, including peak strain (PS), peak systolic strain rate (PSSR) and peak diastolic strain rate (PDSR) in the radial, circumferential and longitudinal directions, were measured. NIDCM (T2DM+) patients were divided into two subgroups based on the HbA1c level (< 7.0% and ≥ 7.0%). The determinants of reduced LV myocardial strain for all NIDCM individuals and NIDCM (T2DM+) patients were assessed using multivariable linear regression analyses.

**Results:**

Compared with normal controls, both NIDCM (T2DM −) and NIDCM (T2DM+) patients exhibited increased LV end-diastolic and end-systolic volume index and decreased LV ejection fraction. LV global strains progressively declined from the normal controls to the NIDCM (T2DM−) group to the NIDCM (T2DM+) group (all p < 0.017), except for radial PDSR and PSSR. Subgroup analysis showed that LV global radial PS and longitudinal PS, PSSR-L and PDSR-L were worse in NIDCM patients with poor glycemic control than in those with good glycemic control (p < 0.017). T2DM was an independent determinant of reduced LV global circumferential PS and longitudinal PS in patients with NIDCM (both p < 0.05). An increased HbA1c level was independently associated with a decreased global radial PS (β = − 0.285, p < 0.01) and longitudinal PS (β = 0.320, p < 0.01) in NIDCM (T2DM+) patients.

**Conclusions:**

T2DM has an additive deleterious effect on LV systolic and diastolic function in NIDCM patients. Among NIDCM patients with T2DM, HbA1c was found to be associated with reduced LV myocardial strain.

## Introduction

Type 2 diabetes mellitus (T2DM) is associated with heart failure (HF) and is an independent risk factor for cardiovascular morbidity and mortality. In T2DM, subclinical cardiac structural and functional abnormalities, including left ventricular (LV) hypertrophy, myocardial fibrosis and stiffness [1], and reduced LV compliance, were found in the absence of coronary artery disease, hypertension, and valvular heart disease [2, 3]. Non-ischemic dilated cardiomyopathy (NIDCM), one of the leading causes of HF, is a life-threatening disease that manifests as an enlargement of the LV or both ventricles with cardiac dysfunction or abnormal loading conditions without any ischemic origin [4, 5]. It is well known that both NIDCM and T2DM can lead to impairment of LV geometry and function, culminating in progressive deterioration of HF and poor outcomes. In particular, recent studies have shown that the prognosis of NIDCM patients with T2DM was worse than that of those without T2DM [6, 7]. Therefore, a better understanding of the additive effect of T2DM on cardiac change in patients with NIDCM is an important step towards achieving health management.

Cardiac magnetic resonance (CMR) is the most accurate technique for assessing cardiac structure and function without the known limitations introduced by echocardiography (e.g., acoustic window limitation, high operator dependency, and low spatial resolution) [8, 9]. CMR feature tracking (CMR-FT) derived myocardial strain is a promising method for quantification of the intrinsic performance of the myocardium. The utility of CMR-FT-derived LV strains in recognizing subclinical and subtle LV changes, identifying patients at higher risk of progressing to the HF stage, and providing detailed information on prognosis have been widely reported [10–12]. However, previous studies investigated the independent effects of T2DM or NIDCM as separate conditions on cardiac structure and function [13, 14], and little is known about the additive effect of T2DM in patients with NIDCM on LV strain using the CMR-FT method. Therefore, the aim of this study was to explore the impact of comorbid T2DM on LV strain by CMR-FT in patients with NIDCM.

## Materials and methods

### Study population

 The study protocol was approved by the Biomedical Research Ethics Committee of our hospital and complied with the Declaration of Helsinki (2013 edition). Informed consent was waived due to the retrospective nature of the research.

**Fig. 1:**
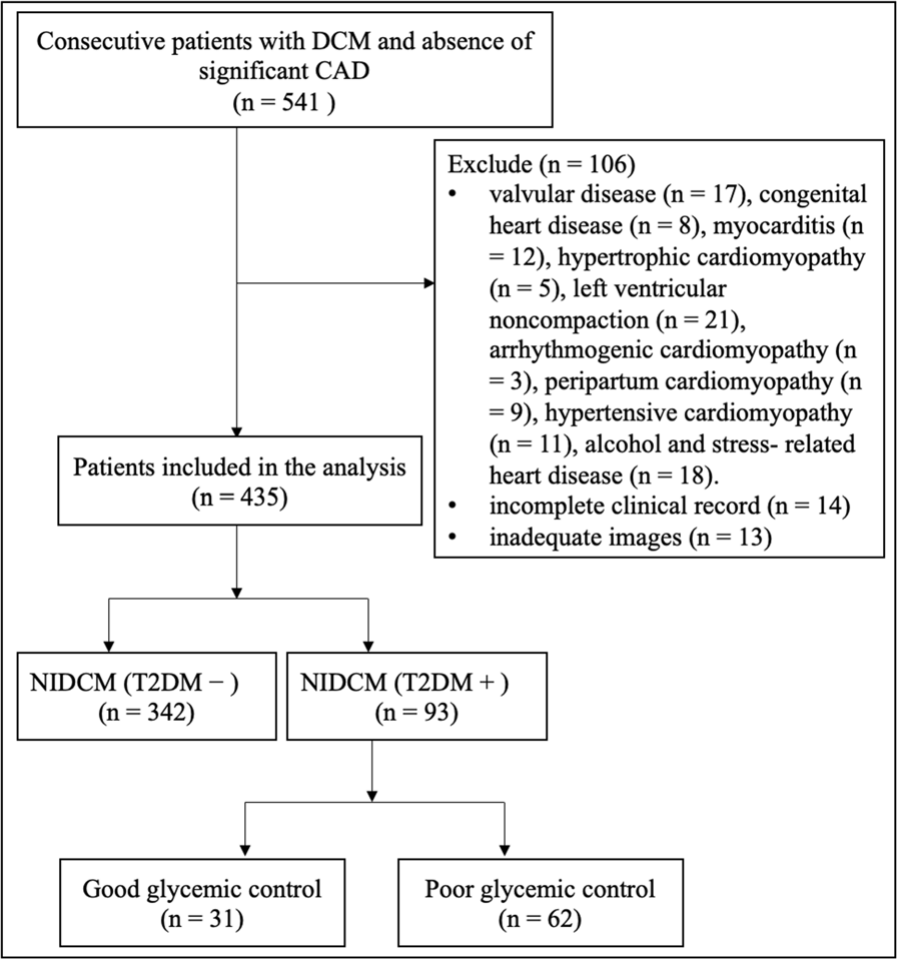
Flow chart of the study. DCM dilated cardiomyopathy, NIDCM non-ischemic dilated cardiomyopathy, T2DM type 2 diabetes mellitus

Initially, we retrospectively enrolled 541 patients with NIDCM who underwent CMR examinations in our hospital from October 2014 to November 2021. The diagnosis of NIDCM was based on the criteria from the World Health Organization/International Society and Federation of Cardiology definition [15]. The inclusion criteria were as follows: (1) impaired LV ejection fraction (LVEF) ≤ 45% assessed with CMR; and (2) the absence of significant coronary artery disease (≥ 50% luminal stenosis of at least one major coronary artery) confirmed by invasive coronary angiography or CT coronary angiography, previous coronary revascularization or myocardial infarction. The exclusion criteria were as follows: (1) HF secondary to other cardiomyopathies or diseases (valvular disease, congenital heart disease, myocarditis, hypertrophic cardiomyopathy, left ventricular noncompaction, arrhythmogenic cardiomyopathy, restrictive cardiomyopathy, constrictive pericarditis, cardiac sarcoidosis, tachycardia-induced cardiomyopathy, peripartum cardiomyopathy, hypertensive cardiomyopathy, alcohol and stress-related heart disease; non-diabetic metabolic or endocrine diseases, severe lung disease; (2) an incomplete clinical record; and (3) inadequate images because of arrhythmia or poor image quality. Finally, 435 NIDCM patients were included in this study. Among them, 93 NIDCM patients presented with T2DM, whereas 342 did not. The diagnosis of T2DM was made according to the current American Diabetes Association guideline recommendations [16]. To evaluate the influence of glycemic control on LV function, NIDCM (T2DM+) patients were categorized as having good glycemic control (HbA1c < 7.0%) or poor glycemic control (HbA1c ≥ 7.0%). In addition, another 80 normal subjects without any other primary diseases or abnormalities on CMR images served as the control group. A detailed flow chart of the present study is presented in Fig. 1.

### CMR protocol

All enrolled subjects underwent CMR on a 3.0T whole-body Siemens MAGNETOM Trio Tim system or a MAGNETOM Skyra system (Siemens Medical Solutions, Erlangen, Germany). A standard ECG-triggering device and breath-hold technique were used to monitor dynamic changes. Cine images such as a stack of short-axis views from the base to apical level and long-axis views (2-chamber, 3-chamber, 4-chamber), were acquired by applying an electrocardiogram-gated balanced steady-state free precession (SSFP) sequence. Typical acquisition parameters were as follows: temporal time = 39.34/40.35 ms, echo time = 1.22/1.20 ms, slice thickness = 8 mm, field of view = 240 × 300/288 × 360 mm^2^, matrix size = 208 × 174/192 × 162 pixels, flip angle = 40°/50°. A dose of 0.2 ml/kg gadobenate dimeglumine (MultiHance; Bracco, Milan, Italy) was intravenously injected at an injection rate of 2.5–3.0 mL/s, followed by a 20 mL saline flush. Late gadolinium enhancement (LGE) images were acquired by a phase-sensitive inversion recovery sequence at 10–15 min after contrast administration (acquisition parameters: TR/TE = 750/1.18; 512/1.24 ms; slice thickness = 8 mm, field of view = 240 × 300/288 × 360 mm^2^, matrix size = 256 × 162/ 256 × 125 pixels, and flip angle = 20°/40°).

### Image analysis

All image analyses were performed by two experienced radiologists (more than three years of experience with CMR) using CVI42 software (Circle Cardiovascular Imaging, Inc., Calgary, Canada). The optimal endocardial and epicardial contours of LV were delineated manually at the end-diastole and end-systole phases from the stack of short-axis cine images. Papillary muscles and moderator bands were diligently excluded. The global cardiac geometry and function parameters, including LV end-diastolic volume (LVEDV), end-systolic volume (LVESV), stroke volume (LVSV), LVEF, and cardiac mass (LV mass), were obtained. Volume and mass measurements were indexed by body surface area (BSA). Additionally, the LV remodelling index was calculated as LV mass divided by LVEDV.

**Fig. 2:**
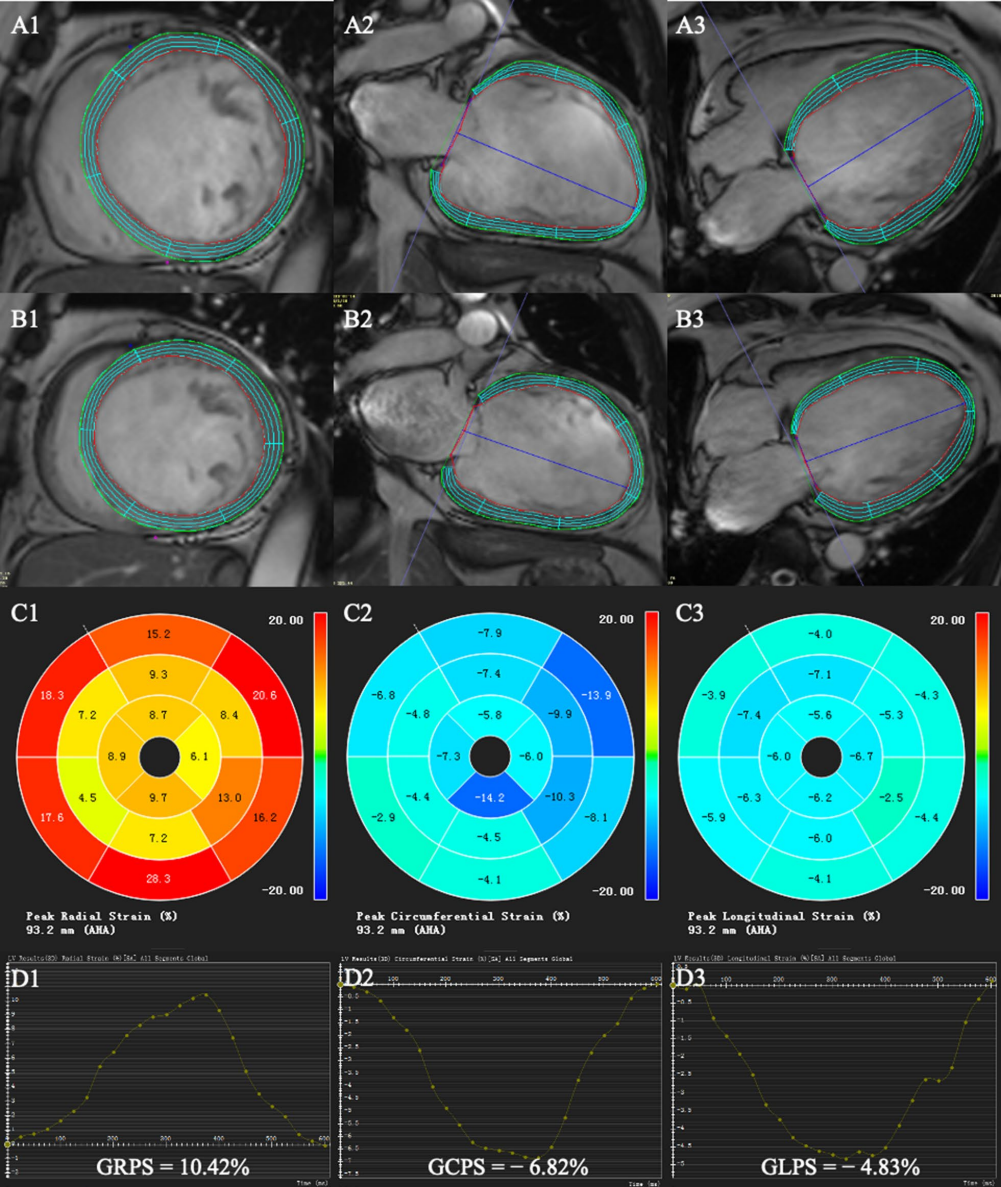
Measurement of LV global strain. Cardiac magnetic resonance tissue tracking in short-axis and long‐axis two‐chamber and four‐chamber cine images at end‐diastole (A1–3) and end-systole (B1–3). The three-dimensional pseudo-color maps of the LV global peak strain (C1–3). The measurement of the LV global peak strain (D1-3).

The LV global strain parameters were quantified on short-axis cine images and long-axis cine images (2-chamber and 4-chamber) using a feature-tracking module. The end-diastolic phase was chosen as a reference phase. To ensure the accuracy of the automated tracking in the subsequent phases, the observer reviewed the tracking performance and performed manual adjustments when needed. Subsequently, three-dimensional feature tracking parameters were obtained: LV global radial peak strain (GRPS), global circumferential peak strain (GCPS), and global longitudinal peak strain (GLPS) and their corresponding peak systolic strain rate (PSSR) and peak diastolic strain rate (PDSR) in the three directions (Fig. 2).

The presence of LGE was visually determined by two experienced operators blinded to the clinical data on post-contrast short-axis and long-axis images. In case of disagreement, a third blinded operator was consulted, and consensus was reached through discussion between the operators.

### Reproducibility

The intra- and interobserver variability were calculated for LV strain parameters in 40 randomly selected patients (including 30 NIDCM patients and 10 normal controls). The intraobserver variability for the strain parameters was measured twice by the same observer (MTS) at an interval of 2 weeks. The interobserver variability was assessed using measurements from the same population by a second independent observer (YL) after 1 week.

### Statistical analysis

All data were analyzed with SPSS statistical software (version 23.0; SPSS Inc., Chicago, IL, USA), R (version 4.0.3; R Foundation for Statistical Computing, Vienna, Austria) with RStudio (Version 1.3.959; RStudio, Boston, Mass), and GraphPad Prism (version 9.0, GraphPad Software Inc., San Diego, CA, USA). Categorical variables are presented as numbers (percentages) and were compared using Fisher’s exact test or the chi-square test, as appropriate. The Shapiro–Wilk test was performed to check for normality of continuous variables. Continuous data are expressed as the mean ± SD for variables with a normal distribution and the median (quartile 25, quartile 75) for those with a nonnormal distribution. Parameters among the NIDCM (T2DM−), NIDCM (T2DM+) and normal control groups were compared by one-way analysis of variance (one-way ANOVA) followed by Bonferroni’s post hoc test (normally distributed variables) or the Kruskal–Wallis rank test (nonparametric variables), as appropriate. The same methods were also utilized for comparison among NIDCM (T2DM+) patients with good and poor glycemic control and the normal control groups. Correlations between LV strains and clinical indices was assessed using Spearman’s analysis. Furthermore, variables with a probability value of less than 0.1 in univariable analysis and an absence of collinearity were included in stepwise multivariable linear regression analysis to identify the independent determinants of LV strain parameters. The intraclass correlation coefficient (ICC) was calculated to evaluate both intra- and interobserver variability. A two-tailed test and a p value of < 0.05 were considered significant.

## Results

### Baseline characteristics

Overall, 515 participants (435 NIDCM and 80 controls) were included in the present study. Of the 435 NIDCM patients, 93 patients (21%) were identified as having T2DM, and the remaining 342 patients (79%) were classified as non-T2DM patients. The main clinical characteristics of the study cohort are summarized in Table 1. Sex, BMI, systolic and diastolic blood pressure, plasma triglycerides, total cholesterol, and high- and low-density lipoprotein were not significantly different between the observed groups. The NIDCM (T2DM+) group was older than both the NIDCM (T2DM−) and control groups (p < 0.017). Compared with control group, the NIDCM groups had lower eGFRs (all p < 0.017). As expected, fasting blood glucose and HbA1c levels were significantly higher in the NIDCM(T2DM+) group than in the NIDCM (T2DM−) group (both p < 0.05). Additionally, the NT-proBNP and troponin T values were significantly higher in the NIDCM (T2DM+) group than in the NIDCM (T2DM−) group (p < 0.05).

**Table 1:** Baseline characteristics of the study population

	Normal controls (n = 80)	NIDCM (T2DM −) (n = 342)	NIDCM (T2DM +) (n = 93)
Age (years)	47.03 ± 12.09	48.89 ± 14.53	55.80 ± 12.07 ^*, §^
Male, n (%)	55 (69)	233 (68)	71 (76)
Systolic BP (mm Hg)	113.59 ± 18.26	113.68 ± 17.87	117.37 ± 20.63
Diastolic BP (mm Hg)	76.38 ± 14.44	74.99 ± 14.84	76.98 ± 13.56
Heart rate (beats/min)	74.15 (65.20, 80.43)	76.00 (66.55, 88.43)	81.75 ± 15.03
BMI (kg/m^2^)	24.05 ± 3.66	23.86 ± 4.78	24.67 ± 3.63
Smoking, n (%)	36 (45)	183 (54)	46 (49)
NYHA class			
(I/II/III/IV)	–	11/85/161/85	2/19/45/27
Diabetes duration (years)	–	–	4.00 (0.30, 10.00)
Fasting blood glucose (mmol/L)	–	5.47 ± 1.29	7.82 (6.12, 10.71) ^§^
HbA1c, (%)	–	5.60 ± 0.54	7.40 (6.80, 8.50) ^§^
TG (mmol/L)	1.41 ± 0.42	1.29 (0.92, 1.81)	1.45 (1.00, 2.04)
TC (mmol/L)	4.33 ± 0.61	4.12 (3.48, 4.86)	4.26 ± 1.11
HDL (mmol/L)	1.43 ± 0.32	1.11 (0.84, 1.41)	1.09 ± 0.34
LDL (mmol/L)	2.54 ± 0.62	2.51 (1.95, 2.98)	2.56 ± 0.86
eGFR (mL/min/1.73 m^2^)	100.01 (92.00, 108.24)	88.61 (74.72, 100.47) ^*^	81.85 ± 21.82 ^*^
NT-proBNP value (pg/mL)	–	1448.00 (628.75, 3402.00)	2473.00 (960.50, 5956.50) ^§^
Troponin T value (ng/L)	–	15.65 (9.88, 29.33)	25.00 (15.60, 44.90) ^§^
*Medical treatment (for DCM), n (%)*			
ACEI/ARB	–	315 (92)	87 (94)
β‐blocker	–	325 (95)	87 (94)
MRA	–	257 (75)	64 (69)
Diuretics	–	212 (62)	62 (67)
Digoxin	–	79 (23)	20 (22)
*Diabetes treatment, n (%)*			
Biguanides	–	–	32 (34)
Sulfonylureas	–	–	17 (18)
a‐Glucosidase inhibitor	–	–	29 (31)
GLP‐1/DPP‐4 inhibitor	–	–	4 (4)
SGLT2 inhibitor	–	–	10 (11)
Insulin	–	–	23 (25)

All values are presented as mean ± SD or n (%) or median (Q1-Q3)

NIDCM non-ischemic dilated cardiomyopathy, T2DM type 2 diabetes diabetes mellitus, BMI body mass index, NYHA New York Heart Association, HbA1c glycated hemoglobin, TG triglycerides, TC total cholesterol, HDL high-density lipoprotein, LDL low-density lipoprotein, eGFR estimated Glomerular Filtration Rate, NT-proBNP N-terminal pro-brain natriuretic peptide, ACEI angiotensin-converting enzyme inhibitor, ARB angiotensin receptor blocker, MRA mineralocorticoid receptor antagonist, GLP-1 glucagon-like peptide-1, DPP-4 dipeptidyl peptidase-4, SGLT2 sodium-glucose co-transporter 2

^*^P < 0.017 vs. normal controls; ^§^P < 0.017 vs. NIDCM (T2DM −) group

### Comparison of CMR-derived indices among NIDCM patients with and without T2DM and normal controls

The CMR findings for the observed groups are presented in Table 2. In contrast to the normal controls, patients who had NIDCM with and without T2DM exhibited an increased LVEDV index and LVESV index, increased LV mass index, and decreased LVSV index and LVEF (all p < 0.017). The NIDCM (T2DM+) group exhibited a higher LV mass index and LV remodelling index, lower LVSV index than the NIDCM (T2DM−) group (p < 0.017), whereas the LVEF was similar between these two groups (p > 0.05). In addition, no significant difference was observed in the presence of LGE between NIDCM patients with and without T2DM (p > 0.05).

**Table 2:** Comparison of CMR‐derived parameters among normal controls, NIDCM patients with and without T2DM

	Normal controls (n = 80)	NIDCM (T2DM −) (n = 342)	NIDCM (T2DM +) (n = 93)
*LV geometry and function*			
LVEDVi (mL/m^2^)	75.29 ± 15.05	163.15 (136.51, 200.08) ^*^	160.58 (135.28, 202.25) ^*^
LVESVi (mL/m^2^)	28.07 ± 8.20	126.64 (94.97, 159.57) ^*^	128.83 (98.16, 163.34) ^*^
LVSVi (mL/m^2^)	47.23 ± 9.81	37.81 (27.55, 48.25) ^*^	34.30 ± 12.82 ^*,§^
LVEF (%)	62.94 ± 6.65	22.79 (16.90, 30.32) ^*^	21.75 ± 9.87 ^*^
LVMI (g/m^2^)	42.73 ± 6.65	70.56 (59.95, 82.50) ^*^	81.25 (61.57, 94.01) ^*, §^
RVEF (%)	56.09 ± 7.06	32.30 (20.42, 46.77) ^*^	27.12 (13.42, 41.11) ^*, §^
LV remodelling index, g/mL	0.57 ± 0.11	0.42 (0.36, 0.50) ^*^	0.46 (0.40, 0.57) ^*, §^
Presence of LGE, n (%)	–	238 (70)	61 (66)
*LV PS (%)*			
GRPS	34.00 (29.98, 40.93)	8.58 (5.91, 12.95) ^*^	6.42 (4.66, 11.17) ^*, §^
GCPS	− 20.61 ± 2.60	− 6.99 (− 9.45, − 5.32) ^*^	− 5.53 (− 8.14, 4.12) ^*, §^
GLPS	− 14.14 ± 2.22	− 5.59 (− 7.34, − 4.23) ^*^	− 4.54 (− 5.89, − 3.22) ^*, §^
*LV PSSR (1/s)*			
Radial	2.10 (1.64, 2.54)	0.59 (0.43, 0.83) ^*^	0.53 (0.36, 0.75) ^*^
Circumferential	− 1.07 (− 1.18, − 0.92)	− 0.44 (− 0.56, − 0.33) ^*^	− 0.38 (− 0.50, − 0.30) ^*, §^
Longitudinal	− 0.79 (− 0.88, − 0.69)	− 0.40 (− 0.52, − 0.31) ^*^	− 0.33 (− 0.47, − 0.26) ^*, §^
LV PDSR (1/s)			
Radial	− 2.50 (− 3.10, − 2.00)	− 0.61 (− 0.93, − 0.45) ^*^	− 0.56 (− 0.90, 0.38) ^*^
Circumferential	1.26 ± 0.24	0.49 (0.36, 0.66) ^*^	0.44 (0.31, 0.60) ^*, §^
Longitudinal	0.91 ± 0.24	0.42 (0.33, 0.55) ^*^	0.36 (0.26, 0.48) ^*, §^

All values are presented as n (%) or median (Q1-Q3). “ − ” indicates the direction of strains. LV left ventricular, EF ejection fraction, LVEDVi left ventricular end-diastolic volume index, LVESVi left ventricular end- systolic volume index, LVSVi left ventricular stroke-volume index, LVMI left ventricular mass index, RVEF right ventricular ejection fraction, LGE late gadolinium enhancement, PS peak strain, GRPS global radial peak strain, GCPS global circumferential peak strain, GLPS global longitudinal peak strain, PSSR peak systolic strain rate, PDSR peak diastolic strain rate, LGE late gadolinium enhancement

^*^P < 0.017 vs. normal group; ^§^P < 0.017 vs. NIDCM (T2DM −) group

Regarding the LV myocardial strains, almost all of the LV global strains progressively decreased from normal controls to the NIDCM (T2DM−) group to the NIDCM (T2DM+) group (all p < 0.017), whereas the PSSR and PDSR in the radial direction showed no significant difference between NIDCM patients with and without T2DM (all p > 0.05).

### Comparison of LV global strains among NIDCM (T2DM+) patients with good and poor glycemic control and normal controls

**Fig. 3:**
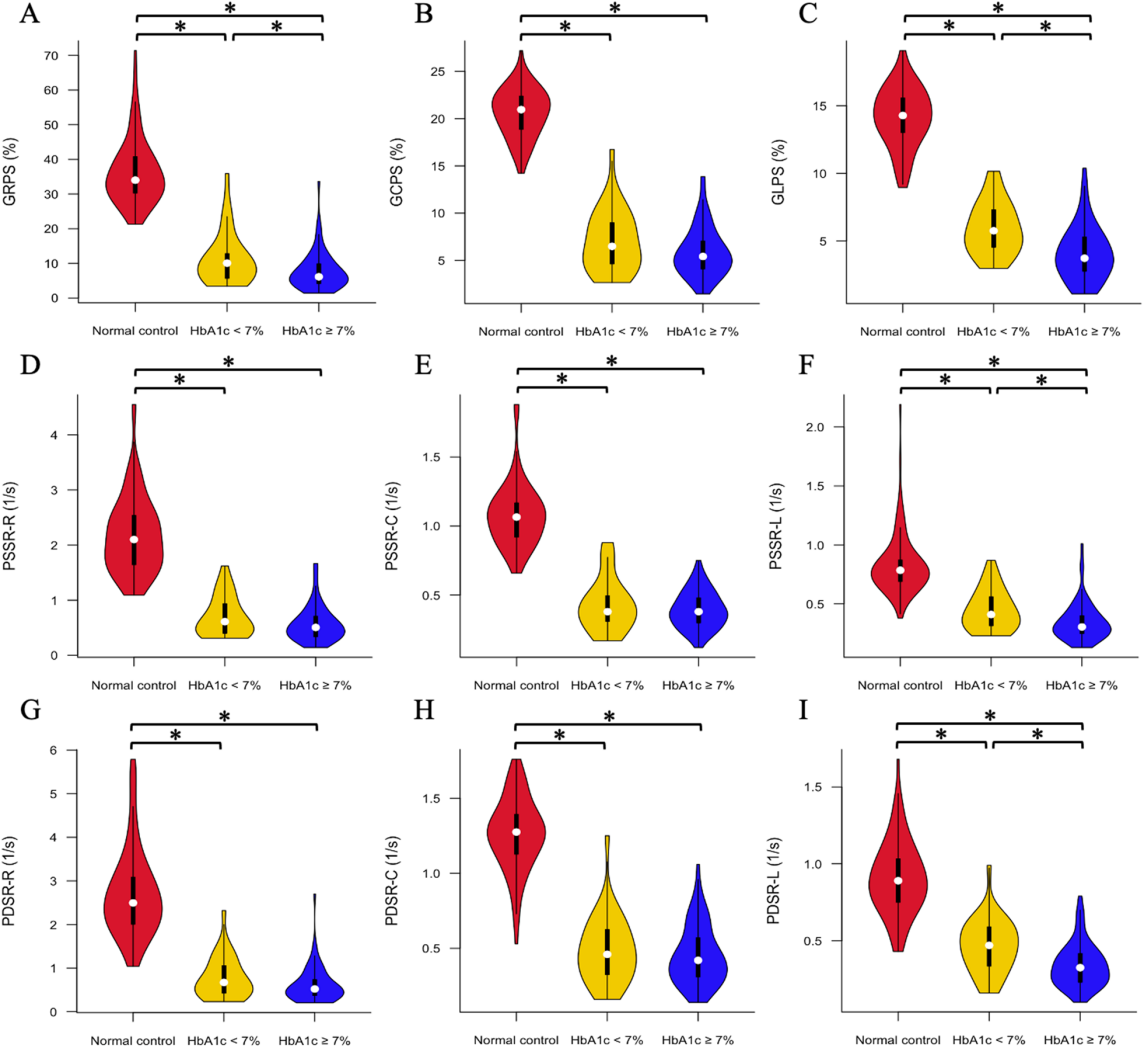
Comparison of LV global strains among NIDCM (T2DM +) patients with good and poor glycemic control and normal controls. The absolute values of LV global strains were used to avoid the influence of directional sign. GRPS global radial peak strain, GCPS global circumferential peak strain, GLPS global longitudinal peak strain, PSSR peak systolic strain rate, PDSR peak diastolic strain rate, R radial, C circumferential, L longitudinal. *p < 0.017

According to the status of glycemic control, NIDCM (T2DM+) patients were divided into two subgroups: good glycemic control (n = 31, HbA1c < 7.0%) and poor glycemic control (n = 62, HbA1c ≥ 7.0%). The CMR-derived LV global strain parameters for the observed groups are shown in Fig. 3. NIDCM (T2DM+) patients with good and poor glycemic control had a lower global PS, PSSR, and PDSR in all three directions than the individuals in the normal control group (all p < 0.017). Additionally, LV GRPS, GLPS, longitudinal PSSR and PDSR were significantly lower in the poor glycemic control group than in the good glycemic control group (all p < 0.017), whereas PS, PSSR, and PDSR in the circumferential direction and radial PSSR and PDSR showed no significant differences between these two groups (all p > 0.017). Moreover, LVEDVi, LVESVi, LVSVi and LVEF were not significantly different between NIDCM (T2DM+) patients with good and poor glycemic control (LVEDVi: 163.48 (122.45, 191.84) mL/m^2^ vs. 172.86 (136.32, 206.97) mL/m^2^; LVESVi: 125.45 (86.05, 157.29) mL/m^2^ vs. 140.43 (103.27, 179.75) mL/m^2^; LVSVi: 38.03 (28.43, 49.86) mL/m^2^ vs. 32.43 (23.09, 40.40) mL/m^2^; LVEF: 24.69% (16.23%, 29.92%) vs. 20.28% (14.21%, 27.31%); all p < 0.017).

### Association between LV global peak strains and clinical variables in NIDCM patients

Univariable and multivariable linear regression analyses were performed to evaluate the independent effect of T2DM on LV peak strains in NIDCM patients (Table 3). After adjusting for confounding factors, the multivariable linear regression analysis showed that T2DM was independently associated with LV GCPS (β = 0.112, p < 0.05) and GLPS (β = 0.139, p < 0.05). Furthermore, heart rate, male sex, and log-transformed NT-proBNP level were independently associated with LV GRPS (β = − 0.160, − 0.127, and – 0.412, all p < 0.05), GCPS (β = 0.158, 0.155 and 0.401, all p < 0.05), and GLPS (β = 0.210, 0.134, and 0.410, all p < 0.05).

**Table 3:** Univariable and multivariable analysis between the LV peak strain and clinical indices in NIDCM patients

	GRPS (%)		GCPS (%)		GLPS (%)	
	Univariable	Multivariable	Univariable	Multivariable	Univariable	Multivariable
	r	β (95%CI)	r	β (95%CI)	r	β (95%CI)
T2DM	− 0.138^*^	–	0.195^*^	0.112 (0.030, 0.194)	0.226^*^	0.139 (0.060, 0.219)
Gender ^#^	− 0.151^*^	− 0.127 (− 0.210, − 0.045)	0.161^*^	0.155 (0.074, 0.236)	0.151^*^	0.134 (0.055, 0.213)
Age	− 0.032	–	0.032	–	0.001	–
Systolic BP	− 0.095^*^	–	− 0.116^*^	− 0.125 (− 0.207, − 0.044)	− 0.028	–
Heart rate	− 0.288^*^	− 0.160 (− 0.246, − 0.075)	0.270^*^	0.158 (0.075, 0.241)	0.340^*^	0.210 (0.129, 0.292)
NYHA	− 0.206^*^	–	0.213^*^	–	0.228^*^	–
HbA1c	− 0.169^*^	–	0.194^*^	–	0.247^*^	–
Fasting plasma glucose	− 0.126^*^	–	0.165^*^	–	0.146^*^	–
HDL	0.152^*^	–	− 0.149^*^	–	− 0.197^*^	–
Log (NT–proBNP) ^&^	− 0.357^*^	− 0.412 (− 0.497, − 0.326)	0.386^*^	0.401 (0.317, 0.485)	0.411^*^	0.410 (0.327, 0.492)
Troponin T	− 0.242^*^	–	0.281^*^	–	0.271^*^	–
eGFR	0.088	–	0.097	–	− 0.071	–
Presence of LGE	− 0.065	–	0.072	–	0.057	–

Abbreviations as in Tables 1, 2

^#^ For gender, we used 0 to represent for female and 1 to represent for male

^&^ NT-proBNP is log-transformed before being included in the regression model

β is adjusted regression coefficient

Factors with P < 0.1 in the univariable analyses were included in the stepwise multiple liner regression model

^*^ P < 0.05

### Independent effect of HbA1c level on LV global peak strains in NIDCM (T2DM+) patients

**Fig. 4:**
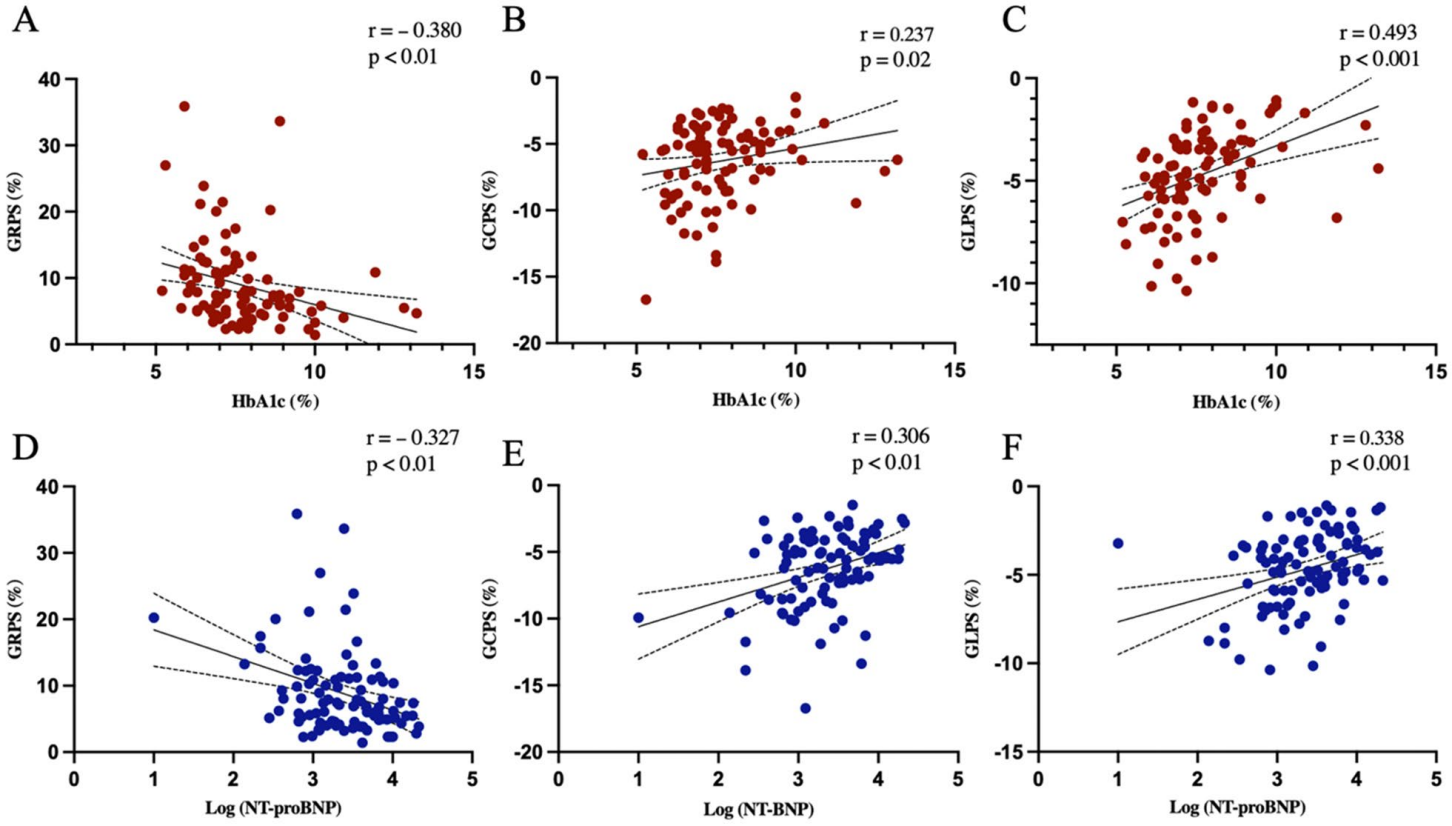
The associations of LV global strains with HbA1c level and NT-proBNP in NIDCM (T2DM +) patients. GRPS global radial peak strain, GCPS global circumferential peak strain, GLPS global longitudinal peak strain

The univariable analysis of NIDCM (T2DM+) patients showed that HbA1c level, together with log-transformed NT-proBNP level, was negatively associated with GRPS (r = − 0.380 and – 0.327, both p < 0.01), and positively associated with GCPS (r = 0.237 and 0.306, both p < 0.05) and GLPS (r = 0.493 and 0.338, both p < 0.001) (Fig. 4). Increasing troponin T value was significantly associated with worsening GRPS (r = − 0.266, p < 0.05) and GCPS (r = 0.297, p < 0.05). In addition, heart rate was negatively associated with GRPS (r = − 0.267, p < 0.05) and positively associated with GLPS (r = 0.408, p < 0.001).

After adjusting for confounding factors, HbA1c remained an independent determinant of impaired GRPS (β= − 0.285, p < 0.001), and GLPS (β = 0.320, p < 0.001). Moreover, the log- transformed NT-proBNP level was found to be an independent determinant of global peak strain in all three directions (GRPS: β= − 0.334, GCPS: β = 0.290, and GLPS: β = 0.227, all p < 0.01) (Table 4).

**Table 4:** Determinants of impaired LV global strains in NIDCM (T2DM +) patients

	GRPS (%)		GCPS (%)		GLPS (%)	
	Univariable	Multivariable	Univariable	Multivariable	Univariable	Multivariable
	r	β (95%CI)	r	β (95%CI)	r	β (95%CI)
Gender ^#^	− 0.158	–	− 0.008	–	0.172	–
Age	0.072	–	0.094	–	− 0.165	–
SBP	− 0.243^*^	–	− 0.204^§^	–	− 0.196^§^	–
Heart rate	− 0.267^*^	–	0.168	–	0.408^*^	0.269 (0.095, 0.442)
NYHA	− 0.188^§^	–	0.215^*^	0.215 (0.015, 0.415)	0.300^*^	–
HbA1c	− 0.380^*^	− 0.285 (− 0.472, − 0.097)	0.237^*^	–	0.493^*^	0.320 (0.146, 0.493)
Fasting plasma glucose	− 0.039	–	0.075	–	0.072	–
Log (NT–proBNP) ^&^	− 0.327^*^	− 0.334 (− 0.522, − 0.147)	0.306^*^	0.290 (0.090, 0.490)	0.338^*^	0.227 (0.051, 0.403)
Troponin T	− 0.266^*^	–	0.297^*^	–	0.130	–
Presence of LGE	0.013	–	0.117	–	0.006	–

Abbreviations as in Tables 1, 2

^#^ For gender, we used 0 to represent for female and 1 to represent for male

^&^ NT-proBNP is log-transformed before being included in the regression model

β is adjusted regression coefficient

Factors with P < 0.1 in the univariable analyses were included in the stepwise multiple liner regression model

^§^ P < 0.1

^*^ P < 0.05

### Intra- and interobserver variability of LV strain parameters

The results of the intra- and interobserver analyses for LV strains are shown in Table 5. The intra- and interobserver agreement was excellent for LV strain parameters (ICC = 0.925–0.977 and 0.915–0.969, respectively).

**Table 5:** Intra‐and interobserver variabilities of LV myocardial strain

	Intraobserver		Interobserver	
	ICC	95%CI	ICC	95%CI
*LV PS*				
Radial	0.925	0.862–0.959	0.922	0.857–0.958
Circumferential	0.962	0.929–0.980	0.956	0.918–0.976
Longitudinal	0.974	0.952–0.986	0.967	0.939–0.982
*LV PSSR*				
Radial	0.945	0.899–0.971	0.915	0.845–0.954
Circumferential	0.927	0.867–0.961	0.934	0.878–0.964
Longitudinal	0.967	0.938–0.982	0.969	0.943–0.984
*LV PDSR*				
Radial	0.957	0.920–0.977	0.936	0.883–0.966
Circumferential	0.935	0.880–0.965	0.931	0.874–0.963
Longitudinal	0.977	0.956–0.988	0.964	0.933–0.981

LV left ventricular; ICC intraclass correlation coefficient, CI confidence interval, PS peak strain, PSSR peak systolic strain rate, PDSR peak diastolic strain rate

## Discussion

This study investigated the combined effects of T2DM on CMR-derived LV deformation in NIDCM patients. The main findings of this study are as follows: (1) NIDCM patients displayed reduced LV deformation, and comorbid T2DM further deteriorated LV strains; (2) after adjustment for confounding factors, T2DM was found to be an independent determinant of reduced LV global circumferential and longitudinal peak strain in NIDCM patients; (3) LV global peak strains declined progressively with the increase in HbA1c in NIDCM patients with T2DM; in addition, HbA1c was an independent determinant of decreased LV global radial and longitudinal peak strains. These findings indicated the deleterious effect of T2DM on LV deformation in patients with NIDCM and emphasized the importance of the glycemic control in NIDCM patients with T2DM.

T2DM is a growing health concern and is associated with HF and increased cardiovascular mortality [17]. The prevalence of HF has been reported to be 15- 44% in diabetes, and HF risk is increased among diabetic patients without coronary heart disease [18, 19]. This association between diabetes and HF reveals that factors other than coronary atherosclerosis may increase HF risk in diabetes [20]. NIDCM is a common primary heart disease that causes HF deterioration in a manner independent of coronary artery disease. Indeed, a previous study reported that the prognosis of NIDCM patients with T2DM was worse than that of those without T2DM, and T2DM was found to be significantly associated with an increased incidence of cardiac events [7, 21]. Similarly, Tanaka et al. also verified that NIDCM patients with T2DM had worse long-term outcomes than those without T2DM, and their study further identified that reduced LV GLPS assessed by speckle tracking echocardiography was associated with worse outcomes in NIDCM patients with T2DM [6]. To explore changes in LV deformation in NIDCM patients with T2DM, we conducted this study and found that comorbid T2DM augmented the impairment of LV strain and strain rate assessed by CMR-FT in NIDCM patients, even the conventional LV function parameter (i.e., LVEF) was similar between these two groups. Further multivariable linear regression analysis showed that T2DM was an independent determinant of LV global circumferential and longitudinal peak strain in patients with NIDCM.

The potential causes for cardiac alterations in NIDCM patients with T2DM are complex. Indeed, DCM is characterized by alterations in myocardial metabolism [22], which manifest as increased myocardial glucose uptake and utilization and decreased fatty acid uptake and utilization [23]. The presence of abnormal glucose tolerance in patients with DCM may exacerbate these changes and reduce myocardial metabolic flexibility in response to increased workload. Myocardial reactive oxygen species production levels have been found to be 5 times higher in DCM patients with diabetes than in idiopathic DCM patients; this may be due to the increase in the production of reactive metabolites caused by elevated intracellular glucose concentrations [24]. Moreover, high heart rate pacing induces a rapid increase in glucose and lactate uptake accompanied by a relative decrease in nonesterified fatty acid uptake [25, 26]. Our multivariable analysis showed that HR is also an independent determinant of LV strains. In addition to direct metabolic derangement-related causes [27], increased intravascular volume burden may play a role in LV function change. Since glucose is also an osmotically active molecule, hyperglycemia can lead to intravascular volume overload, resulting in increased strain on the heart and activation of the natriuretic peptide system. These mechanisms increase cardiac pressure in a synergistic manner and are even more pronounced in patients with NIDCM, which itself is also associated with hypervolemia [28]. Likewise, our study verified that NT-proBNP levels were significantly higher in NIDCM patients with T2DM than in those without T2DM, and the log transformed NT-proBNP level was an independent determinant of LV strains in NIDCM patients with T2DM, which means that NIDCM patients with comorbid T2DM have a more pronounced cardiac load and that this elevated natriuretic peptide level was associated with impaired LV function. In terms of histological analysis, a prior study observed greater impairment of myocardial relaxation, increased myocardial fibrosis, and mitochondrial degeneration of the myocardium in NIDCM patients with DM than in those without DM [21]. Frustaci et al. also demonstrated more pronounced mitochondrial damage and myofibrillolysis in DM-complicated NIDCM [24]. These findings may underlie the exacerbation of LV function in NIDCM patients with T2DM.

HbA1c, a biomarker of long-term blood glucose concentrations that represents the average blood glucose levels over the previous 8–12 weeks, is widely used to assess the status of glycemic control for patients with diabetes. Jia et al. found that elevated blood glucose levels were significantly associated with incident HF events [29]. Each 1% increase in HbA1c is linked to an 8% increase in the risk of HF in T2DM [29]. In addition to HF, poor glycemic control is an independent determinant of LV myocardial strains for T2DM patients [30]. More importantly, the relationship between HbA1c and HF has been reported in NIDCM patients as well. A previous study reported that poor glycemic control may increase the risk of subsequent HF events in NIDCM patients [31]. In the current study, our data showed that an increased HbA1c is associated with decreased LV strains, and this association is more prominent in NIDCM patients with T2DM. Multivariable linear regression analysis showed that HbA1c was an independent determinant of LV global radial and longitudinal peak strain in NIDCM patients with T2DM. Similarly, Ikeda et al. found that hyperglycemic is a risk factor for the progression of concomitant abnormal LV relaxation during follow-up, resulting in poor prognosis in patients with NIDCM [31]. Another study confirmed this change in myocardial relaxation by cardiac catheterization in patients with DM-complicated NIDCM [21]. A previous study demonstrated that poor glycemic control is closely related to severe endothelial dysfunction [32]. Furthermore, hyperglycemia-induced oxidative stress, autonomic deficiency, inflammation, abnormalities in calcium homeostasis, apoptosis, and interstitial fibrosis are also responsible for cardiac remodelling and dysfunction [2, 3, 33]. Since HbA1c is an independent determinant of LV global function, considerable attention should be given to the status of glycemic control in NIDCM patients with T2DM.

Additionally, our data found that male sex was an independent determinant of LV strains in patients with NIDCM. A previous study suggested that male sex is an important risk factor for HF among DCM patients. Men with DCM had higher apoptosis-related protein expression than women, and the incidence of myocardial fibrosis was higher in men than women [34, 35]. Meanwhile, male sex also adversely affects the expression of diabetic cardiomyopathy in T2DM [36]. The sex difference observed in DCM and T2DM is primarily attributed to the effects of sex hormones.

## Limitation

The current study had several limitations. First, this was a retrospective single-centre study, so there may be some selection bias in the results. Second, the influence of medication on NIDCM patients was not assessed. Third, this study was a cross-sectional study, and long-term outcomes and follow-up examinations evaluating the evolution of LV function were not available, so further studies are needed in this regard. Finally, although the presence of LGE was assessed in the present study, the extent and pattern of LGE were not evaluated; we aim to accomplish this in our future research.

## Conclusions

In patients with NIDCM, T2DM had an additive deleterious effect on LV myocardial strain. In addition, the HbA1c level was associated with decreased LV function in NIDCM patients with T2DM, which emphasizes the importance of glycemic control in NIDCM patients.

## Data Availability

The datasets used and/or analyzed during the current study are available from the corresponding author on reasonable request.

## Abbreviations

T2DM: Type 2 diabetes mellitus.
NIDCM: Non-ischemic dilated cardiomyopathy
LV: Left ventricular
PS: Peak strai
PSSR: Peak systolic strain rate
PDSR: Peak diastolic strain rate
GRPS: Global radial peak strain
GCPS: Global circumferential peak strain
GLPS: Global longitudinal peak strain
HbA1c: Glycated hemoglobin
HF: Heart failure
CMR: Cardiac magnetic resonance
LVEF: Left ventricular ejection fraction
LVEDV: Left ventricular end-diastolic volume
LVESV: Left ventricular end-systolic volume
LVSV: Left ventricular stroke volume
BSA: Body surface area
ICC: Interclass correlation coefficient
eGFR: Estimated glomerular filtration rate
NT-proBNP: N-terminal pro-brain natriuretic peptide
LGE: Late gadolinium enhancement
